# Adipose Mesenchymal Extracellular Vesicles as Alpha-1-Antitrypsin Physiological Delivery Systems for Lung Regeneration

**DOI:** 10.3390/cells8090965

**Published:** 2019-08-23

**Authors:** Elia Bari, Ilaria Ferrarotti, Dario Di Silvestre, Pietro Grisoli, Valentina Barzon, Alice Balderacchi, Maria Luisa Torre, Rossana Rossi, Pierluigi Mauri, Angelo Guido Corsico, Sara Perteghella

**Affiliations:** 1 Department of Drug Sciences, University of Pavia, Viale Taramelli 12, 27100 Pavia, Italy; 2 Center for Diagnosis of Inherited Alpha1-antitrypsin Deficiency, Department of Internal Medicine and Therapeutics, Pneumology Unit, IRCCS San Matteo Hospital Foundation, University of Pavia, 27100 Pavia, Italy; 3 Institute for Biomedical Technologies, F.lli Cervi 93, 20090 Segrate, Milan, Italy; 4 PharmaExceed S.r.l., Piazza Castello, 19, 27100 Pavia, Italy

**Keywords:** mesenchymal secretome, mesenchymal extracellular vesicles, mesenchymal exosomes, mesenchymal microvesicles, alpha-1-antitrypsin, lung diseases, anti-elastase

## Abstract

Accumulating evidence shows that Mesenchymal Stem/Stromal Cells (MSCs) exert their therapeutic effects by the release of secretome, made of both soluble proteins and nano/microstructured extracellular vesicles (EVs). In this work, for the first time, we proved by a proteomic investigation that adipose-derived (AD)-MSC-secretome contains alpha-1-antitrypsin (AAT), the main elastase inhibitor in the lung, 72 other proteins involved in protease/antiprotease balance, and 46 proteins involved in the response to bacteria. By secretome fractionation, we proved that AAT is present both in the soluble fraction of secretome and aggregated and/or adsorbed on the surface of EVs, that can act as natural carriers promoting AAT in vivo stability and activity. To modulate secretome composition, AD-MSCs were cultured in different stimulating conditions, such as serum starvation or chemicals (IL-1β and/or dexamethasone) and the expression of the gene encoding for AAT was increased. By testing in vitro the anti-elastase activity of MSC-secretome, a dose-dependent effect was observed; chemical stimulation of AD-MSCs did not increase their secretome anti-elastase activity. Finally, MSC-secretome showed anti-bacterial activity on Gram-negative bacteria, especially for *Klebsiella pneumoniae*. These preliminary results, in addition to the already demonstrated immunomodulation, pave the way for the use of MSC-secretome in the treatment of AAT-deficiency lung diseases.

## 1. Introduction

In the last few years, Mesenchymal Stem Cells (MSCs) have been largely employed in regenerative medicine, as they demonstrated capability in limiting inflammation, reprogramming immune cells, and activating endogenous repair pathways [1,2]. Accumulating evidence shows that MSCs exert their therapeutic effects by the release of secretome, made of both soluble factors (cytokine, chemokine, and growth factors) and nano/microstructured extracellular vesicles (EVs) [3]. The secretome isolated from MSCs derived from bone marrow, Worton jelly, umbilical cord, and adipose tissue, has been tested in different pre-clinical models of lung injury and showed to effectively regulate the pathophysiological responses of the damaged tissues. Improvement in lung function, increased cell survival, restoration of lung architecture, reduction of fibrosis, increased alveolarization, and a general reduction in the inflammatory response were the most common observations [4]. The use of secretome for therapeutic purposes, instead of a cell, leads to advantages in terms of safety (secretome is considered virtually non-tumorigenic, less immunogenic than cells, and its vascular administration does not cause vascular clotting) and technological issues (secretome can be sterilized, handled, and stored more easily) [5,6]. In this context, recently, we defined a scalable Good Manufacturing Practice (GMP)-compliant production process for freeze-dried MSC-secretome (lyo-secretome) [7].

Alpha-1-antitrypsin (AAT) is a 52 kDa single-chain glycoprotein abundantly produced in the liver by hepatocytes and, in small amounts, by macrophages, monocytes, neutrophils, and by the bronchial epithelium [8]. Once released into the blood circulation, it enters the tissues, especially in the lungs, and protects them from being damaged by proteolytic enzymes, such as trypsin, elastase, and protease-3 [8]. In Europe, it is estimated that about 1 in 2000 people have AAT deficiency, an inherited genetic disorder, totaling about 370,000 people with low levels of AAT in plasma (15–25 mg/dl, with respect to the diagnostic reference levels of 90–200 mg/dL) (http://orpha.net, last access: 20/11/2018). Pathophysiology of the disorder lies in variants in the *SERPINA1* gene that is located on the long arm of chromosome 14 (14q31–32·3) and that encodes for AAT. More than 100 alleles have been identified and among them, approximately 30 are correlated with clinical implications [9]. The most common is mutation Z (approximately 95% of the cases) and it results from the substitution of lysine for glutamic acid at position 342 of the *SERPINA1* gene. This mutation allows spontaneous polymerization of AAT with consequent accumulation of polymers within the hepatocyte that causes a decrease in the amount of circulating AAT. AAT deficiency manifests as pulmonary emphysema, liver cirrhosis (the consequence of the AAT polymer accumulation) and, rarely, as the skin disease panniculitis [10]. Lung disease is thought to occur because of the lack of protection provided by AAT in inactivating neutrophil elastase, a proteolytic enzyme released from inflammatory cells in the lungs as a defense mechanism to destroy damaged cells and bacteria. Moreover, AAT appears to exert important anti-inflammatory and immunomodulatory effects in the lungs, including reduction of Toll-like receptor expression, reduction of neutrophil adherence to the endothelium, and reduction of secreted pro-inflammatory cytokines [11,12]. The generated protease–anti-protease imbalance and the pro-inflammatory environment lead to degradation of elastin and other basal membrane and matrix components in the lung parenchyma, and consequently, development of the chronic obstructive pulmonary disease (COPD) and emphysema [13,14]. Environmental factors, especially cigarette smoking and dust exposure, showed to accelerate the progression of this condition [10,15,16].

The current standard of care for patients affected by AAT deficiency-associated pulmonary emphysema is the augmentation therapy by weekly intravenous infusion of pooled human plasma purified AAT (the weekly dose usually recommended is 60 mg/kg). The rationale is based on raising the serum and lung levels of AAT and, therefore, on restoring the balance of proteases and anti-proteases. The drugs available on the market are Prolastin^®^ [17], Prolastin C^®^ [17], Glassia^®^ [18], Zemaira^®^ [19], and Aralast^®^ [20], which show minor chemical differences as a result of the methods used during purification [21]. No recombinant protein drug has been approved to date. The augmentation therapy appears to be safe, and overall, notwithstanding the lack of definitive evidence, findings suggest that it helps to reduce the progression of emphysema [22]. Promising alternative treatments include PEGylation of AAT, which consists of the conjugation to the protein of polyethylene glycol (PEG) polymer chains to increase its blood half-life [23], gene therapy [24], promotion of AAT hepatic secretion by drugs [25,26], and therapies aimed at reducing lung injury (antioxidants) or at promoting lung re-epithelialization (all-trans-retinoic acid) [27]. 

In this paper, for the first time to our knowledge, we revealed by a proteomic investigation that MSC lyo-secretome contains AAT and we then investigated the potential employment of MSC-secretome in the treatment of AAT deficiency-associated lung diseases. The reduced level of AAT induces an excess of protease activity, that leads to the damage and destruction of the pulmonary tissue, predisposing it to a greater risk of bacterial infections and triggering the inflammatory response. Therefore, to be active in such a pathological context, MSC-secretome should (i) inhibit the excess of protease activity, (ii) protect against the onset of bacterial infections, and (iii) regulate the inflammatory process. In this regard, in the first part of the manuscript, we performed a molecular characterization of freeze-dried MSC-secretome (lyo-secretome) at proteomic level highlighting the proteins involved in protease/antiprotease balance, acute phase response, and in the response to bacteria. Then, we investigated if AAT is released by AD-MSCs as free-soluble protein or packaged into EVs and we tested different stimulating conditions in order to modulate the MSC-secretome composition in relation to the therapeutic needs, and thus to increase its in vitro anti-elastase properties. Finally, we also investigated lyo-secretome antimicrobial properties that could be fundamental for pulmonary infection associated with AAT-deficiency.

## 2. Materials and Methods

### 2.1. Materials

All reagents used for cell cultures were purchased from Euroclone (Milan, Italy). Acetone, AAT, bovine serum albumin, collagenase, dexamethasone, IL-1β, mannitol, Nile Red, Phosphate Buffered Saline, and phosphatidylcholine were obtained from Sigma-Aldrich (Milan, Italy). AD-MSCs were supplied by Tissue Bank and Tissue Therapy Unit, Emergency and Acceptance Department, ASST Niguarda Hospital, Milan, Italy, in accordance with the Declaration of Helsinki, and the protocol was approved by the Ethics Committee of ASST Grande Ospedale Metropolitano Niguarda (Milan, Italy) (Ref. 12 November 2009). For this study, AD-MSCs were chosen because of greater ease of access and collection than harvesting bone marrow stem cells, and for the presence of many validated and known protocols for their isolation, expansion, and secretome collection.

### 2.2. Lyo-Secretome Preparation

#### 2.2.1. Cell Culture and Secretome Collection

Lyo-secretome was prepared according to a previously reported method with slight modifications [7,28,29]. Adipose-derived MSCs (AD-MSCs) were harvested from adipose tissues [30,31] collected from three female patients (mean age was 47 ± 4) undergoing abdominoplasty surgery after informed consent. Donors with septicemia or extensive infections, Creutzfeld–Jacobs disease, type B and C hepatitis, syphilis, HIV, viral or unknown neurological diseases, and malignant tumors were excluded. Upon reaching sub-confluence AD-MSCs (at P3) were cultured in DMEM/F12 minimal medium (plus 1% (*v/v*) penicillin/streptomycin and 1% (*v/v*) amphotericin B) for 48 h; conditioned media collected after 9 and 24 h were pooled as well as conditioned media collected at 33 and 48 h. Then, AD-MSCs were detached with trypsin-EDTA and tested to evaluate cell viability and the concordance with all the requirements needed for clinical use in terms of identity (according to the International Society for Cellular Therapy [32]) and sterility (according to Eu. Ph. 9.0, 2.6.27). DNA was isolated from each AD-MSC line using a commercial extraction kit (DNA IQ System; Promega, Milan, Italy). Sequencing all coding exons (II–V) of the *SERPINA1* gene (RefSeq: NG_008290) was performed as reported previously [33], using the CEQ 8800 genetic analysis System (Beckman Coulter, Milan, Italy). Sequence analysis detected the presence of mutation F (p.R247C c.739C>T rs28929470) in heterozygous fashion in one of the three cell lines. Due to the well-known dysfunctional properties of the F variant of the *SERPINA1* gene [34], this AD-MSC cell line was removed from further analysis. The other two AD-MSC cell lines did not report pathological (deficient or dysfunctional) mutations.

#### 2.2.2. MSC-Secretome Concentration, Purification, and Lyophilization

Cell debris and apoptotic bodies were eliminated from the conditioned media by centrifugation at 3500× *g* for 10 min. Then, supernatants were collected and concentrated to 0.5 × 10^6^ cell equivalents per mL (calculated dividing the total cell number and the mL of concentrated and purified supernatant) by tangential flow filtration (KrosFlo^®^ Research 2i system, Spectrum Laboratories, Milan, Italy). A filtration module with Molecular Weight Cut Off (MWCO) of 5 kDa (Spectrum Laboratories, Milan, Italy) with a superficial area of 235 cm^2^ was chosen to retain both free soluble factors and EVs produced by AD-MSCs. Finally, the concentrated conditioned medium was diafiltered using sterilized ultrapure water. During all the ultrafiltration steps, according to the manufacturer’s instruction, the transmembrane pressure did not exceed 10 psi and the shear rate of the feed stream was maintained between 2000 and 6000 s^−1^. Mannitol was dissolved into concentrated and purified secretome (final concentration of 0.5% *w/v*); the resulting solution was frozen at −80 °C and freeze-dried (Christ Epsilon 2–16D LSCplus) at 8 × 10^−1^ mbar and −50 °C for 72 h. The obtained lyo-secretome was stored at −20 °C until use (2 months). Each mg of lyo-secretome corresponds to 0.1 × 10^6^ cell equivalents (calculated dividing the total cell number used for the production and the obtained milligrams of lyo-secretome). All the concentration, purification, and lyophilization procedures were GMP-compliant.

#### 2.2.3. MSC-Secretome Fraction Separation and Freeze-Drying

Conditioned media, obtained as reported in Section 2.2.1, were centrifuged at 3500× *g* for 10 min, and diafiltered but not concentrated by tangential flow filtration. Subsequently, the diafiltered secretome fraction, having molecular weight (MW) higher than 5 kDa, was progressively ultrafiltered using filtering modules having 300 and 100 kDa MWCO to separate (i) the EV-enriched fraction (having MW ≥ 300 kDa); (ii) the protein soluble fraction at low molecular weight (having MW between 5 and 100 kDa), and (iii) the protein soluble fraction at high molecular weight (having MW between 100 and 300 kDa). Each isolated fraction was concentrated by ultrafiltration to 0.5 × 10^6^ cell equivalents per mL, added with 0.5 *w/v* mannitol, frozen at −80 °C, and subjected to the lyophilization process as previously reported.

### 2.3. Lyo-Secretome and Secretome Fractions Characterization

A full characterization, as reported below, was performed on all the freeze-dried samples. 

#### 2.3.1. Total Protein Content

Total protein content was assessed by BCA Protein Assay Kit from Thermo Fisher Scientific (Milan, Italy), according to manufacturer’s instructions. To each sample (or standard) the working reagent solution was added in a 1:1 ratio; the mixture was then incubated at 37 °C for 2 h and the absorbance was measured at 562 nm with a microplate reader (Synergy HT, BioTek, United Kingdom). The protein concentration was extrapolated from a concentration graph against absorbance obtained from standard protein solutions (Bovine Serum Albumin), using a third-degree polynomial equation, with R^2^ = 0.99.

#### 2.3.2. Total Lipid Content 

Lipids were quantified by Nile Red assay as reported by [7,35]. A Nile Red stock solution (3.14 M) was prepared and stored at 4 °C avoiding light exposure until use. The stock solution was diluted to 0.0314 M in PBS before the use and 10 µL of it were incubated with 90 µL of samples; after 5 min the relative fluorescence was measured by Synergy HT at fixed wavelengths (530/25 excitation and 645/40 emission). The lipid concentration was extrapolated from a concentration graph against fluorescence obtained from standard phosphatidylcholine solutions, using a third-degree polynomial equation, with R^2^ = 0.99.

#### 2.3.3. Alpha-1-Antitrypsin Dosage

AAT was measured by a rate immune nephelometric method (Immage 800 Immunochemistry System, Beckman-Coulter, Fullerton, CA, USA) in the samples before and after the lyophilization process. The measurement was performed in solutions before lyophilization without further dilution and in dried powder diluted in sterile water according to respective protein content, by using the modified method described by [36]. 

#### 2.3.4. Physical-Chemical Characterization

The physical-chemical properties of all the freeze-dried products have been investigated as reported in [7]. In detail, the IR spectra in transmittance mode were obtained by a Spectrum One Perkin-Elmer spectrophotometer equipped with a MIRacle™ ATR device in the spectral region of 650–4000 cm^−1^. Differential Scanning Calorimetry (DSC) analysis was performed by Mettler STARe system equipped with a DSC821e Module and an Intracooler device (Julabo FT 900) for sub-ambient temperature. The residual humidity was investigated by Thermo Gravimetric Analysis (TGA) with a Mettler STARe system equipped with a TGA/DSC1. 

#### 2.3.5. Network Analysis

Already available MSC-secretome protein profile (of both free-soluble factors and EVs) was evaluated at the system level as previously reported [7]. Briefly, a protein-protein interaction (PPI) network was built by combining proteins secreted by human AD-MSCs and *Homo sapiens* PPI data retrieved from the major repositories; only interactions experimentally determined were considered. To retrieve and visualize PPIs, as well as to process the reconstructed networks, Cytoscape and its plugins were used [37]. Specifically, String [38] and Pesca [39] plugins were used to retrieve PPIs, UniProt database (www.uniprot.org) and Cytoscape plugin BINGO 2.44 [40] were used to evaluate the most represented GO terms; as for BINGO, *Homo sapiens* organism, hypergeometric test, Benjamini-Hochberg FDR correction and a significance level ≤ 0.001 were set. In addition, reconstructed networks were analyzed at the topological level and Cytoscape plugin CentiScaPe [41] was used to calculate centrality indices of each node; in particular, betweenness, centroid, and node degree were calculated.

### 2.4. Stimulated-Secretome Preparation and Characterization

To modulate secretome composition and possibly enhance its therapeutic activity, upon reaching sub-confluence, AD-MSCs were cultured in DMEM/F12 minimal medium for 48 h not supplemented or supplemented with dexamethasone (DEX, 1 µg/mL) and/or IL-1β (10 ng/mL). After 9, 24, and 33 h, the supernatants were removed and replaced with fresh DMEM/F12 minimal medium not supplemented or supplemented with DEX (1 µg/mL) and/or IL-1β (10 ng/mL). After 48 h from the beginning of the process, the supernatant was collected and added to that collected previously. For each group (CTR48, DEX, IL-1β and DEX + IL-1β), supernatants were concentrated by ultrafiltration to 0.5 × 10^6^ cell equivalents per mL, added with 0.5 *w/v* mannitol, frozen at −80 °C, and subjected to the lyophilization process as previously reported. On each freeze-dried sample protein, lipid and AAT content were determined and the physical-chemical characterization was performed as previously reported.

#### Evaluation of AAT Gene Expression in AD-MSCs

Expression of *SERPINA1* transcripts in AD-MSCs was evaluated at the end of the serum starvation (control) and stimulating conditions by conventional reverse transcription-polymerase chain reaction amplification (RT-PCR). Total RNA extraction was performed using the RNeasy mini kit (Qiagen, Hilden, Germany) on QIAcube (Qiagen, Hilden, Germany). Afterwards, cDNA synthesis was carried out by reverse transcription using ThermoScript TM RT-PCR (Invitrogen, Carlsbad, CA). Previously designed forward and reverse primers located in exons exon 2 and 5 were used [42]. PCR was performed on 2 μL of cDNA template and 100 ng of each primer. The amplification conditions were 50 °C for 45′ and 85°C for 5′. PCR products were separated by electrophoresis and visualized on gel-red stained 2% agarose gels. The expression of AAT transcripts was quantitatively analyzed by SYBR Green. All experiments were performed in triplicate and data were managed using the Applied Biosystems 7500 software v2.0 (Thermo Fisher Scientific (Milan, Italy). Relative expression was calculated by using the comparative Ct method and obtaining the fold-change value (ΔCt). The ΔCt value of each sample was calculated using actin, as an endogenous control gene. Briefly, the qPCR mastermixPlus for SYBR Green I (EuroGenTec, Seraing, Belgium) was used and qPCR reactions were carried out as follows: 95 °C for 10 min for enzyme activation; then 40 cycles at 95 °C for 1 s and at 60 °C for 1 min, followed by plate read.

### 2.5. In Vitro Anti-Elastase Activity 

The inhibitory effect of all freeze-dried samples on the elastase enzyme was evaluated according to the method described by Chlapanidas and colleagues [43] with some modifications. Samples were tested at different concentrations after re-suspension in an aqueous reaction solution (final concentrations 5, 10, 50, and 100 mg/mL). The anti-elastase activity of standard AAT (Sigma Aldrich, Milan, Italy) (purified from human plasma and freeze-dried), was also tested to make comparison with lyo-secretome. The enzyme porcine pancreatic elastase (PPE) was diluted in phosphate buffer at pH 6.8 at a concentration of 0.5 units/mL. The substrate N-succinyl-tri-l-alanine-4-nitroanilide was solubilized in TRIS-HCl buffer pH 8 to a final concentration of 0.41 mM. Then, 12.5 µL of elastase solution were incubated with 50 µL of substrate solution and 12.5 µL of sample aqueous solution. A total of 12.5 µL of PBS and 50 µL of TRIS-HCl were added to obtain a final mixture volume of 150 µL and reaction kinetics were evaluated at 410 nm wavelength with a UV–vis microplate reader (Synergy HT, Milan, Italy). The assay was carried out in triplicate. Reaction mixture without samples was used as a negative control. Epigallocatechin gallate (EGCG), used as a positive control, was solubilized in the aqueous reaction solution to obtain a final concentration of 0.6 mg/mL. Elastase inhibition percentage was calculated using the following formula:

% inhibition = (A − B)/A × 100(1)

where A is the optical density (OD) of the negative control and B is the OD of the tested solution. 

### 2.6. Antibacterial Activity Measurements

The antimicrobial activity of lyo-secretome obtained from 48 h serum starvation was evaluated against the following reference bacterial strains (commonly responsible of airway infections): *Staphylococcus aureus* ATCC 6538, *Staphylococcus aureus* MRSA ATCC 33591, *Klebsiella pneuomoniae* ATCC 13883, and *Pseudomonas aeruginosa* ATCC 10356. Before testing, bacteria were grown overnight in Tryptone Soya Broth (TSA, Oxoid, Basingstoke, UK) at 37 °C. The cultures were centrifuged at 224× *g* for 20 min to separate microorganisms from culture broth and then washed with purified water. Washed cells were re-suspended in Dulbecco’s PBS and OD was adjusted to 0.3, corresponding approximately to 1 × 10^8^ colony forming units (CFU)/mL at 650 nm wavelength. The antimicrobial activity was evaluated in the presence and in the absence of lyo-secretome previously solubilized in water (final concentrations: 2 and 6 mg/mL). In particular, aliquots (1 mL) of the microbial suspensions were added to 1 mL of the lyo-secretome solution to obtain a 1:1 solution. For each microorganism employed, a suspension was prepared in PBS without lyo-secretome solution and used as a control. Then, 1:1 solution and controls were incubated at 37 °C. Viable microbial counts were evaluated after contact for 5 and 24 h with the lyo-secretome and in control suspensions; bacterial colonies were enumerated in TSA after incubation at 37 °C for 24 h. The microbicidal effect (ME value) was calculated for each test organism and contact times according to the following equation [44,45]:

ME = logN_c_ – logN_d_, (2)

where N_c_ is the number of CFU of the control microbial suspension and N_d_ is the number of CFU of the microbial suspension in presence of lyo-secretome.

### 2.7. Statistical Analysis

Raw data were processed using STATGRAPHICS XVII (Statpoint Technologies, Inc., Warrenton, VA, USA). A linear generalized Analysis of Variance model (ANOVA) was used, followed by Fisher’s least significant difference (LSD) procedure to estimate the differences between the means. In detail, the protein and lipid content of each batch was analyzed considering batch number as a fixed factor and protein/lipid amount as the response variable. The anti-elastase results were analyzed considering the time and type of sample as fixed factors and the concentration as a covariate. The enzymatic kinetics of anti-elastase activity were elaborated with a model of Michaelis-Menten kinetics y = (Vmax × *x*) / (Km + *x*), where y is the absorbance at time *x*, Km is the moment in which the activity is equal to half the maximum, and Vmax is the maximum speed of the enzyme [43]. The curve parameters were calculated with the Graph-Pad Prism software version 8 for the Windows^®^ platform. For each curve, Vmax and Km were analyzed with an ANCOVA model, considering the sample as a fixed factor and the constant as a covariate. The differences between the groups were analyzed with the LSD test for multiple comparisons. Antimicrobial activity results were processed by a linear generalized Analysis of Variance model (ANOVA) considering the bacterial strain, the lyo-secretome concentration, and the exposure time as fixed factors. Statistical significance was fixed at *p* < 0.05.

## 3. Results

In this work, the method of Bari and colleagues was employed to obtain secretome from AD-MSCs by serum starvation and to formulate it into pharmaceuticals combining ultrafiltration and lyophilization [7]. In-process analyses were performed at the end of each critical step (serum starvation, ultrafiltration, and lyophilization) in order to define the compliance with the quality criteria needed to continue in the next steps (or to start again if the process went badly and the quality criteria were not complied). In detail, at the end of serum starvation, MSCs were tested to evaluate cell viability and the concordance with all the requirements needed for clinical use in terms of identity and sterility; by our experience (more than 160 pilot batches of lyo-secretome produced in 4 years) this ensures that proteins and EVs were released by cells. At the end of the ultrafiltration, proteins, lipids and AAT were dosed to demonstrate that the concentration and purification phases successfully occurred and to verify the reproducibility of the final product. Finally, in addition to the total protein, AAT and lipid content, the pharmaceutical quality of all the freeze-dried samples was defined by means of a physical-chemical characterization: (i) the absorbance bands detected by FTIR confirmed the simultaneous presence of both proteins and EVs in the freeze-dried products; (ii) the DSC thermal profile showed that mannitol, the cryoprotectant, resulted stable in the β form, and the absence of any thermal effect related to eutectic melting or glass transition events indicates that the lyophilization process successfully occurred; the TGA curve showed a maximum mass loss always below 1% in the temperature range between 50 and 80 °C due to dehydration (data not shown). 

**Figure 1:**
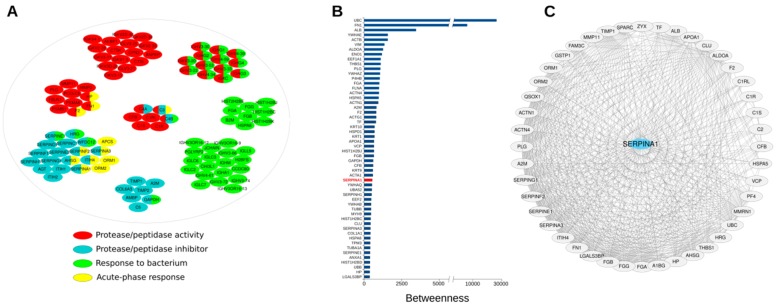
(**A**) Proteins secreted by human Adipose-Derived Mesenchymal Stem/Stromal Cells (AD-MSCs) and grouped in four functional categories including protease/peptidase activity, protease/peptidase inhibitor activity, response to bacteria, and acute-phase response (see Table S1); in addition, proteins belonging to a functional category were further split, where possible, by protein families. (**B**) Topological evaluation of the network (290 nodes and 3836 interactions) reconstructed by combining proteins secreted by human AD-MSCs and *Homo sapiens* protein-protein interaction (PPI) data [7]. Proteins in the graph showed betweenness, centroid, and degree values above the average calculated on the whole network, thus are considered hubs; the complete list (290 proteins) and the corresponding topological parameters (betweenness, centroid, and degree) are shown in Table S2. (**C**) Proteins secreted by human AD-MSCs experimentally identified [7] and annotated as *SERPINA1* interactors.

In our previous work, we characterized the proteomic content of the lyo-secretome and our analyses led to the identification of 348 proteins clustered in 16 network modules [7]. Based on a further functional classification, performed considering the therapeutic properties secretome should ideally possess to treat lung diseases caused by AAT-deficiency, we now report that about 30% (101 proteins) of the secreted proteins belong to four functional categories: protease/peptidase activity (46 proteins), protease/peptidase inhibitors (27 proteins), response to bacterium (46 proteins), and acute-phase response (12 proteins) (Figure 1, Table S1). Most proteins resulted involved in response to bacterium and protease/antiprotease balance, which include alpha-1-antitrypsin (*SERPINA1*). By fully connected PPI network (290 proteins and 3836 interactions) reconstructed by starting from all the identified proteins (348 proteins) [7], we found that alpha-1-antitrypsin (*SERPINA1*) showed betweenness, centroid, and degree values above the average calculated on the whole network, thus resulting as a candidate hub protein (Figure 1B, Table S2). Hub proteins occupy specific network positions and are characterized by a high number of interactions, thus are considered as organizing regulatory molecules, functionally capable of holding together communicating proteins, as well as crucial to maintaining functionally and coherence of signalling mechanisms [41,46]. In fact, we experimentally identified 44 proteins annotated as directly interacting with alpha-1-antitrypsin *SERPINA1*, including other SERPINS, complement and coagulation factors, and metalloproteases (Figure 1C).

**Figure 2:**
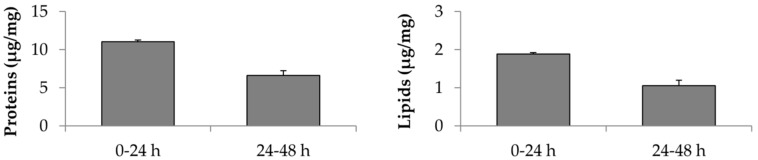
Total protein and lipid content of freeze-dried secretome collected between 0–24 and 24–48 h of serum starvation; mean values ± standard deviation, *n* = 3.

In Figure 2 the amount of proteins and lipids contained in freeze-dried MSC-secretome collected between 0–24 and 24–48 h of serum starvation is reported. Increasing the serum starvation time (48 h vs. 24 h), AD-MSCs continue to release proteins and lipids. In detail, cells produced 59.9% more proteins and 55.81% more lipids. In Table 1 the AAT amount dosed into three different lyo-secretome batches produced after 48 h of serum starvation are reported.

**Figure 3:**
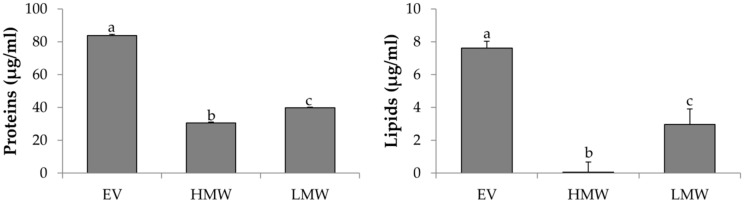
Total proteins and lipids dosed into MSC-secretome fractions. Mean values ± standard deviation, *n* = 3. Different letters (a, b, c) indicate significant differences between the means (*p* < 0.05), whereas the same letter indicates no significant difference (*p* > 0.05).

In a second experiment, the whole secretome was progressively fractioned to separate the EV-enriched fraction (EV, MW > 300 kDa) from the low molecular weight protein fraction (LMW, 5 < MW < 100 kDa) and from the high molecular weight protein fraction (HMW, 100 < MW < 300 kDa). The aim was to investigate if AAT is secreted by cells as a free-soluble factor or encapsulated/adsorbed on EVs. The amounts of proteins and lipids contained into EV were significantly higher compared to the LMW and HMW fractions. Of note, the protein content reported for the EV fraction is, probably, underestimated: as no vesicle digestion was performed, only the proteins exposed on the lipidic layer, and not the ones internalized, may have been detected. While the HMW fraction revealed no lipids, the LMW fraction retained high amounts of lipids, about half of the EV fraction (Figure 3). Interestingly, the amount of proteins for both the LMW and HMW fractions was similar, but significant amounts of AAT were revealed only for the LMW fraction (Table 2). 

**Figure 4:**
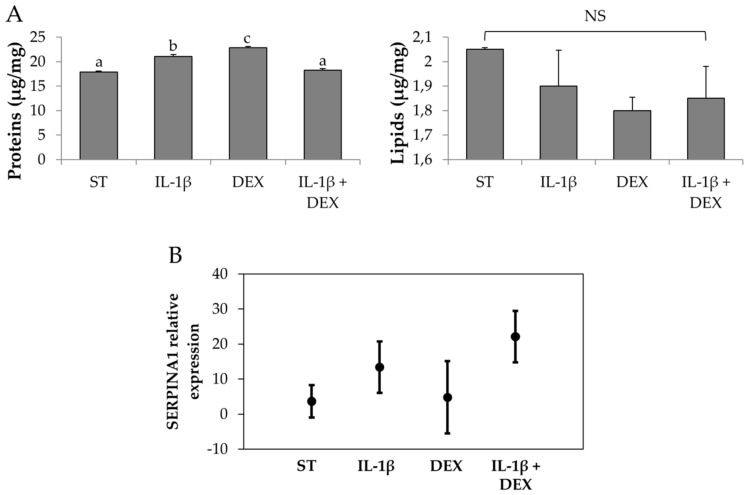
(**A**) Total proteins and lipids dosed into freeze-dried secretome collected after AD-MSC stimulation with different conditions (serum starvation (ST) and/or IL-1β, dexamethasone (DEX) and IL-1β + DEX). Mean values ± standard deviation, *n* = 3. Different letters (a, b, c) indicate significant differences between the means (*p* < 0.05), whereas the same letter indicates no significant difference (*p* > 0.05). (**B**) *SERPINA1* relative expression as a function of the stimulating conditions. Multifactor ANOVA, mean values ± least significant difference (LSD), *n* = 3.

The next intention was to investigate if the exposure to stimulating conditions (e.g., pro-inflammatory cytokines) could increase the amount of proteins, lipids, and AAT produced. In addition to the increasing in the serum starvation time (48 h vs. 24 h, Figure 2), the treatment with DEX or IL-1β increased the amount of proteins produced by AD-MSCs with respect to the control (48 h of serum starvation, ST) but not the amount of lipids (*p* = 0.083) (Figure 4A). In detail, after the DEX stimulation, the amount of proteins was significantly higher (*p* < 0.05). The combined stimulation with IL-1β and DEX, instead, did not increase the proteins produced in a significant manner with respect to the control (ST). The expression of AAT transcripts, evaluated by PCR, was definitely increased after ST and after treatment with IL-1 β and DEX (Figure 4B). 

**Figure 5:**
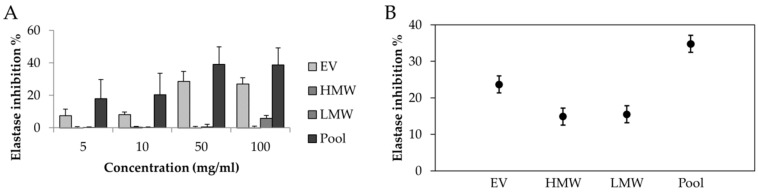
(**A**) Anti-elastase activities of freeze-dried MSC-secretome fractions; mean values ± standard deviation, *n* = 3. (**B**) Results of average elastase inhibition % as a function of the secretome fraction. Multifactor ANOVA, mean values ± LSD, *n* = 3.

The activities of elastase inhibitions exhibited by the MSC-secretome fractions are shown in Figure 5. EV fraction was the only that showed good anti-elastase activity; the LMW fraction showed low anti-elastase activity (5.85% ± 1.7%) only at the highest dose considered (100 mg/mL). The statistical analysis showed that, overall, the elastase inhibition % was significantly higher for the pool ( *p* < 0.05).

**Figure 6:**
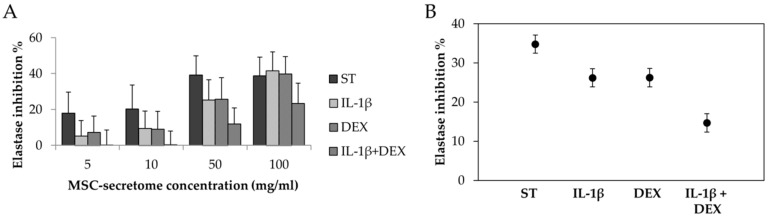
(**A**) Anti-elastase activities of freeze-dried MSC-secretome obtained stimulating AD-MSCs with IL-1β and/or DEX; mean values ± standard deviation, *n* = 3. (**B**) Results of average elastase inhibition % as a function of the different stimulating conditions. Multifactor ANOVA, mean values ± LSD, *n* = 3.

Figure 6 reports the mean anti-elastase % activities of MSC-secretome obtained by different stimulating conditions. A dose-dependent increase of anti-elastase % inhibition was revealed increasing the concentration/mg of freeze-dried secretome and thus the total protein and AAT content. All the batches exhibited a good anti-elastase activity, and at the highest concentration (100 mg/mL) the inhibition rates of ST secretome, DEX secretome, IL-1β secretome, and DEX + IL-1β secretome were 46.17% ± 13.546%, 39.97% ± 9.609%, 41.77% ± 10.446%, and 32.74% ± 15.196%, respectively (mean values ± standard deviation, *n* = 3); however, their values were lower than positive control (epigallocatechin gallate, 91.40% at a concentration of 0.6 mg/mL). The anti-elastase activity percentage of mannitol, the cryoprotectant, was 3.58% ± 0.978% at the highest concentration tested (100 mg/mL). 

The anti-elastase activity of standard AAT (purified from human plasma) was also evaluated to compare with lyo-secretome. The purity of commercial AAT used in this experiment (≥95%) is comparable to those reported in commercial drugs available for augmentation therapy in patients with AAT deficiency. A set of concentrations similar to AAT dosed into lyo-secretome was selected (from 7.5 to 120 µg/mL). The elastase inhibition % values were: 0.75 ± 0.405 for 7.5 µg/mL, 13.34 ± 3.869 for 15 µg/mL, 23.98 ± 5.136 for 30 µg/mL, 74.57 ± 3.019 for 60 µg/mL, and 97.02 ± 1.760 for 120 µg/mL (mean values ± standard deviation, *n* = 3).

The enzymatic kinetics of anti-elastase activity were elaborated with a model of Michaelis-Menten kinetics to extrapolate the Vmax and Km values, as reported in Table 3. 

For the EV fraction and for the secretome obtained from AD-MSCs by serum starvation, the Km was significantly higher compared to the negative control. Instead, the Vmax of secretome samples was not significantly different from the negative control. 

**Figure 7:**
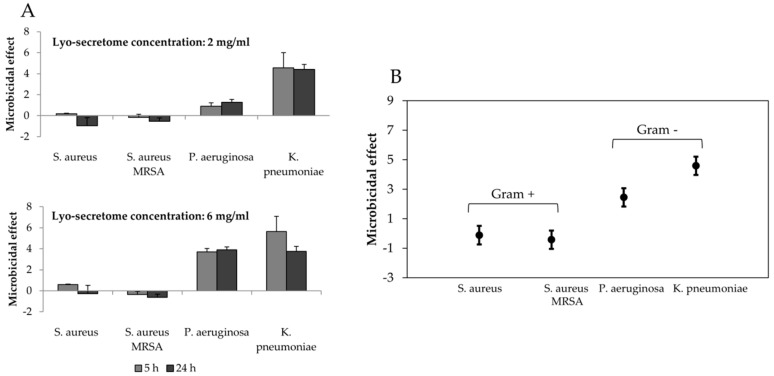
(**A**) Microbicidal effect (ME) of lyo-secretome on the different bacterial strains at 2 and 6 mg/mL after 5 and 24 h of contact time. Mean values ± standard deviation, *n* = 3. (**B**) Lyo-secretome microbicidal effect as a function of the bacterial strain. Multifactor ANOVA, mean values ± LSD, *n* = 3.

Finally, the microbicidal effect (ME) of lyo-secretome was evaluated against bacterial strains belonging to Gram-positive (*S. aureus, S. aureus* MRSA) and Gram-negative (*K. pneumoniae, Ps. aeruginosa*). The relatively logarithmic lowering of the bacterial population was calculated after 5 and 24 h of contact with lyo-secretome using a standard procedure [47,48]. The results showed that the antimicrobial effect of lyo-secretome was already high after 5 h of contact for Gram-negative bacteria and negligible for Gram-positive (Figure 7), for both the concentrations tested. The statistical analysis revealed that the ME effect was significantly influenced by lyo-secretome concentration (*p* < 0.05) but not by the time (*p* = 0.096). The greater effect was observed for *K. pneumonia* reaching respectively about 5.64 and 4.57 logarithmic units lowering of the bacterial population after the two contact times (at a lyo-secretome concentration of 6 mg/mL).

## 4. Discussion

Paracrine factors and EVs secreted by MSCs have been proposed as an alternative to cell therapies. In this work, we proved, by a proteomic investigation, that AD-MSC-secretome contains AAT with high anti-elastase in vitro activity. AAT is a glycoprotein mainly produced in the liver by hepatocytes and, in small amounts, also by macrophages, monocytes, neutrophils, and tissues as bronchial epithelium and pancreatic islets [8]. No reports are present to date about AAT production by AD-MSCs and no author proposed the use of MSCs or their secretome for the treatment of AAT-deficiency pulmonary diseases; only Baligar and colleagues observed the improvement of liver pathology caused by AAT deficiency after MSCs transplantation [49]. It has to be noted that for patients with AAT deficiency, probably, MSCs will not express AAT, even if this not confirmed in the literature. A specific investigation is required, and if this aspect is confirmed, an allogenic use of MSC-secretome will be needed.

Considering the secretome separation into fractions, we demonstered that AAT is mainly contained into the EV fraction with MW higher than 300 kDa. Smaller amounts of AAT were also dosed into the protein soluble fraction at low molecular weight, while it was practically absent in the protein soluble fraction at the high molecular weight. Considering that AAT is a glycoprotein with a MW of 52 kDa, we supposed that in the protein fraction at the low molecular weight it is present in the monomeric form, not aggregated. Instead, the AAT dosed into the EV fraction is, probably aggregated and/or adsorbed on the surface of the vesicles. AAT association with EVs could provide benefits in terms of improved in vivo stability and activity, exploitation of endogenous mechanisms for uptake, intracellular trafficking, and subsequent delivery in recipient cells. In fact, EVs entail several advantages as drug delivery vehicles [50]; among all, we consider their ability to protect cargo from degradation [51], stability in the blood circulation, ability to overcome natural barriers [52,53], and intrinsic cell targeting properties. AAT associated with EVs was also the more active one inhibiting the elastase enzyme, even if its activity was lower compared to the whole secretome. 

Many authors in the literature demonstrated that MSCs can be “educated” by different stimulating conditions to produce the best secretome in relation to the therapeutic use [54,55,56,57]. Therefore, we supposed that cells could be treated also to increase the production of AAT and thus the antiprotease activity of their secretome, with considerable advantages for patients suffering from lung diseases caused by AAT-deficiency. We demonstrated that, in addition to serum starvation, the presence of a chemical stimulus (IL-1β and/or DEX) modified the amount of proteins produced by AD-MSCs and effectively increased the expression of the gene encoding for AAT. A dose-dependent effect was observed testing the anti-elastase activity of freeze-dried secretome obtained by different stimulating conditions. However, the secretome obtained with the only serum starvation, without chemical stimuli, was the one more active inhibiting the elastase. Therefore, we supposed that the chemical stimulation changed the composition of the secretome but did not enhance its anti-elastase activity. We supposed that cells, probably, need more time to convert mRNA resulting from increased gene expression into protein (AAT) able to increase the anti-elastase properties of MSC-secretome. Therefore, an increased stimulation dose or time must be tested, as well as new stimulating conditions. According to another aspect, following chemical stimulation, AD-MSCs can produce proteins with similar, antagonist, and/or synergistic effects with respect to AAT. This could lead to a non-increase in the overall anti-elastase activity (detectable by our in vitro analytical method), even if AAT increases. 

Km and Vmax values gave more information about the elastase inhibition mechanisms: AAT inhibited the elastase enzyme with a non-competitive mechanism. This is in agreement with the literature: Stoller and colleagues described that AAT distorts and inactivates the elastase molecule by squeezing it [58], and therefore it does not compete with the substrate for binding to the active site of the enzyme. Interestingly, MSC-secretome obtained by serum starvation and the EV fraction, instead, inhibited the elastase enzyme by a competitive mechanism, as the Km is increased but the Vmax is not reduced with respect to the negative control [59]. The different mechanism of inhibition suggests that the anti-elastase activity of MSC-secretome is due also to other protease inhibitors, as confirmed by proteomic and PPI network analysis. 

The proteomic analysis also revealed the presence of proteins involved in the response to bacteria. This is in agreement with a recent review of the literature which evidenced that MSCs exert antimicrobial effects both through direct and indirect mechanisms, largely mediated by the secretion of antimicrobial peptides and proteins [60]. Our results demonstrated that MSC-secretome has antimicrobial activity on Gram-negative bacteria; in particular, for *K. pneumoniae* the values of ME are sufficiently high to reach a full antimicrobial effect at the concentration investigated comparable to the action of a disinfectant [44]. The greater resistance of Gram-positive bacteria is probably due to the different composition of the cell wall. In fact, Gram-positive bacteria present a relatively thick and continuous cell wall (thickness 20–80 nm) consisting mainly of peptidoglycan. Conversely, Gram-negative bacteria feature a thinner peptidoglycan layer (thickness 5–10 nm) surrounded by an outer phospholipidic membrane. This membrane contains, among other components, a group of protein-channels called porins, and allows the passage of small hydrophilic antibiotics and metabolites into the cell. 

The anti-protease activity of MSC-secretome, in addition to its anti-microbial activity and its anti-inflammatory properties, demonstrated both by us [29] and others [61,62,63,64,65], lay a solid foundation on the rational use of secretome for the treatment of various pulmonary diseases. In fact, high protease activity is a common feature shared by a wide range of pulmonary pathologies, such as idiopathic pulmonary fibrosis, cystic fibrosis, and emphysema [66]. Frequently, an excess of protease activity leads to the damage and destruction of the pulmonary tissue, predisposing it to a greater risk of bacterial infections and triggering the inflammatory process. Interestingly, the small size of soluble proteins and EVs of secretome allows a more flexible administration with respect to the cells. Therefore, pulmonary administration is possible, thus localizing the action of the secretome directly in the lung, reducing doses and frequency of administration. This aspect is more facilitated by the transformation of MSC-secretome into a pharmaceutical product that we performed. Importantly, during our isolation process, the EV integrity is favored by both the lower stresses generated by the ultrafiltration process, compared to the high shear forces of ultracentrifugation [67], and by the cryoprotectant (mannitol) which prevent EVs from collapsing during freezing. The maintenance of the whole phospholipidic layer is an important aspect, as it protects EV cargo (including the easily-degradable genetic material) from degradation and it preserves the ability to exploit endogenous mechanisms for cell recipient uptake. In fact, the distribution to lung and the subsequent cellular uptake are the main steps for secretome EVs or soluble proteins to exert their therapeutic effect. 

However, many challenges still need to be addressed before the administration of aerosolized secretome, as we recently discussed [4]. In particular, secretome must be administered as powder particles or aerosol droplets with appropriate density, shape, charge, and surface properties for bronchiole and alveolar deposition. Moreover, EVs have to cross the mucous layer that covers the airways. Even if they are facilitated in this goal by their small size, the inclusion of excipients in the formulation will be needed to enhance mucus penetration. In this regard, mannitol, that we used as a cryoprotectant to formulate lyo-secretome, showed to reduce the solids content, surface tension, contact angle, and viscoelastic mucus properties [68]. Overcoming this challenge will be a huge and significant advance with profound clinical improvements in the treatment of AAT deficiency lung diseases.

## 5. Conclusions

In this work, by proteomic investigation, we proved that lyo-secretome contains AAT, 72 other proteins involved in protease/antiprotease balance, and 46 proteins involved in the response to bacteria. AAT is present both in the soluble fraction of secretome and aggregated and/or adsorbed on the surface of EVs, that can act as natural carriers promoting AAT in vivo stability and activity. In AD-MSCs, the expression of the gene encoding for AAT was increased by the presence of a chemical stimulus (IL-1β and/or DEX). A dose-dependent effect was observed in vitro testing the anti-elastase and anti-bacterial activities of the freeze-dried secretome. These results, in addition to the already demonstrated immunomodulation, pave the way for the use of MSC-secretome in the treatment of AAT-deficiency lung diseases.

## Figures and Tables

**Table 1:** Alpha-1-antitrypsin (AAT) content of three lyo-secretome batches produced after 48 h of serum starvation. Results are expressed as µg of protein per mg of sample and mathematically converted to nM, based on a molecular weight of 52 kDa; mean values ± standard deviation, *n* = 3.

Lyo-Secretome Batch	AAT (µg/mg)	AAT (nM)
1	0.78 ± 0.06	15.0 ± 1.1
2	0.83 ± 0.08	16.0 ± 1.5
3	1.24 ± 0.09	23.8 ± 1.7

**Table 2:** AAT content of freeze-dried secretome fractions. Results are expressed as µg of protein per mg of the freeze-dried sample and mathematically converted to nM, based on a molecular weight of 52 kDa; mean values ± standard deviation, *n* = 3.

MSC-Secretome Fraction	AAT (µg/mg)	AAT (nM)
EV	1.37 ± 0.25	26.3 ± 4.8
HMW	0.16 ± 0.05	3.1 ± 0.9
LMW	1.38 ± 0.64	26.5± 12

**Table 3:** Vmax and Km values for each sample analyzed. Multifactor ANOVA, mean values ± standard error, *n* = 9. Different letters (a, b, c) indicate significant differences between the means (*p* < 0.05), whereas the same letter indicates no significant difference (*p* > 0.05).

Sample	Km		Vmax	
	Mean	St. err.	Mean	St. err.
EV	20.2^a^	2.534	0.878^a^	0.03708
LMW	10.2^b^	2.534	0.827^a^	0.03708
HMW	9.85^b^	2.534	0.818^a^	0.03708
ST	20.6^a^	2.534	0.894^a^	0.03708
DEX	15.6^b^	2.534	0.861^a^	0.03708
IL-1β	15.6^b^	2.534	0.860^a^	0.03708
IL-1β + DEX	11.7^b^	2.534	0.876^a^	0.03708
AAT	11.2^b^	2.196	0.569^b^	0.03214
CTR +	20.5^a^	2.158	0.0484^c^	0.03158
CTR -	9.96^b^	2.158	0.856^a^	0.03158

## Supplementary Materials

The following are available online at https://www.mdpi.com/2073-4409/8/9/965/s1.

## References

[1] Phinney D.G., Pittenger M.F. (2017). Concise Review: MSC-Derived Exosomes for Cell-Free Therapy. Stem Cells.

[2] De Girolamo L., Lucarelli E., Alessandri G., Avanzini M.A., Bernardo M.E., Biagi E., Brini A.T., D’Amico G., Fagioli F., Ferrero I. (2013). Mesenchymal Stem/Stromal Cells: A New “Cells as Drugs” Paradigm. Efficacy and Critical Aspects in Cell Therapy. Curr. Pharm. Des..

[3] Crivelli B., Chlapanidas T., Perteghella S., Lucarelli E., Pascucci L., Brini A.T., Ferrero I., Marazzi M., Pessina A., Torre M.L. (2017). Mesenchymal stem/stromal cell extracellular vesicles: From active principle to next generation drug delivery system. J. Control. Release.

[4] Bari E., Ferrarotti I., Torre M.L., Corsico A.G., Perteghella S. (2019). Mesenchymal stem/stromal cell secretome for lung regeneration: The long way through “pharmaceuticalization” for the best formulation. J. Control. Release.

[5] Giebel B., Lambros K., Verena B. (2017). Clinical potential of mesenchymal stem/stromal cell-derivedextracellular vesicles. Stem Cell Invest..

[6] Gimona M., Pachler K., Laner-Plamberger S., Schallmoser K., Rohde E. (2017). Manufacturing of Human Extracellular Vesicle-Based Therapeutics for Clinical Use. Int. J. Mol. Sci..

[7] Bari E., Perteghella S., Di Silvestre D., Sorlini M., Catenacci L., Sorrenti M., Marrubini G., Rossi R., Tripodo G., Mauri P. (2018). Pilot Production of Mesenchymal Stem/Stromal Freeze-Dried Secretome for Cell-Free Regenerative Nanomedicine: A Validated GMP-Compliant Process. Cells.

[8] Fregonese L., Stolk J. (2008). Hereditary alpha-1-antitrypsin deficiency and its clinical consequences. Orphanet J. Rare Dis..

[9] Tonelli A.R., Brantly M.L. (2010). Augmentation therapy in alpha-1 antitrypsin deficiency: advances and controversies. Ther. Adv. Respir. Dis..

[10] Stoller J.K., Aboussouan L.S. (2005). alpha 1-antitrypsin deficiency. Lancet.

[11] Jonigk D., Al-Omari M., Maegel L., Mueller M., Izykowski N., Hong J., Hong K., Kim S.-H., Dorsch M., Mahadeva R. (2013). Anti-inflammatory and immunomodulatory properties of alpha 1-antitrypsin without inhibition of elastase. Proc. Natl. Acad. Sci. USA.

[12] Churg A., Wang X., Wang R.D., Meixner S.C., Pryzdial E.L.G., Wright J.L. (2007). α1-Antitrypsin Suppresses TNF-α and MMP-12 Production by Cigarette Smoke–Stimulated Macrophages. Am. J. Respir. Cell Mol. Boil..

[13] Gramegna A., Aliberti S., Confalonieri M., Corsico A., Richeldi L., Vancheri C., Blasi F. (2018). Alpha-1 antitrypsin deficiency as a common treatable mechanism in chronic respiratory disorders and for conditions different from pulmonary emphysema? A commentary on the new European Respiratory Society statement. Mult. Respi. Med..

[14] Greene C.M., Marciniak S.J., Teckman J., Ferrarotti I., Brantly M.L., Lomas D.A., Stoller J.K., McElvaney N.G. (2018). alpha 1-Antitrypsin deficiency (vol 2, 16051, 2016). Nat. Rev. Dis. Primers.

[15] Wang F., Ni S.-S., Liu H. (2016). Pollutional haze and COPD: etiology, epidemiology, pathogenesis, pathology, biological markers and therapy. J. Thorac. Dis..

[16] Mehta A.J., Miedinger D., Keidel D., Bettschart R., Bircher A., Bridevaux P.-O., Curjuric I., Kromhout H., Rochat T., Rothe T. (2012). Occupational Exposure to Dusts, Gases, and Fumes and Incidence of Chronic Obstructive Pulmonary Disease in the Swiss Cohort Study on Air Pollution and Lung and Heart Diseases in Adults. Am. J. Respir. Crit. Care Med..

[17] Stocks J.M., Brantly M.L., Wang-Smith L., Campos M.A., Chapman K.R., Kueppers F., Sandhaus R.A., Strange C., Turino G. (2010). Pharmacokinetic comparability of Prolastin-C to Prolastin in alpha₁-antitrypsin deficiency: a randomized study. BMC Clin. Pharmacol..

[18] Sandhaus R.A., Stocks J., Rouhani F.N., Brantly M., Strauss P. (2014). Biochemical Efficacy and Safety of a New, Ready-to-Use, Liquid Alpha-1-Proteinase Inhibitor, GLASSIA (Alpha(1)-Proteinase Inhibitor (Human), Intravenous). Copd-J. Chron. Obst. Pulmon. Dis..

[19] Stocks J.M., Brantly M., Pollock D., Barker A., Kueppers F., Strange C., Donohue J.F., Sandhaus R. (2006). Multi-Center Study: The Biochemical Efficacy, Safety and Tolerability of a New α 1 -Proteinase Inhibitor, Zemaira. COPD: J. Chronic Obstr. Pulm. Dis..

[20] Mordwinkin N.M., Louie S.G. (2007). Aralast: An α1-protease inhibitor for the treatment of α-antitrypsin deficiency. Expert Opin. Pharmacother..

[21] Kolarich D., Turecek P.L., Weber A., Mitterer A., Graninger M., Matthiessen P., Nicolaes G.A., Altmann F., Schwarz H.P. (2006). Biochemical, molecular characterization, and glycoproteomic analyses of ?1-proteinase inhibitor products used for replacement therapy. Transfusion.

[22] Teschler H. (2015). Long-term experience in the treatment of α1-antitrypsin deficiency: 25 years of augmentation therapy. Eur. Respir. Rev..

[23] Cantin A.M., Woods D.E., Cloutier D., Dufour E.K., LeDuc R. (2002). Polyethylene Glycol Conjugation at Cys232Prolongs the Half-Life ofα1 Proteinase Inhibitor. Am. J. Respir. Cell Mol. Boil..

[24] Flotte T.R., Brantly M.L., Spencer L.T., Byrne B.J., Spencer C.T., Baker D.J., Humphries M. (2004). Phase I Trial of Intramuscular Injection of a Recombinant Adeno-Associated Virus Alpha 1-Antitrypsin (rAAV2-CB-hAAT) Gene Vector to AAT-Deficient Adults. Hum. Gene Ther..

[25] Burrows J.A.J., Willis L.K., Perlmutter D.H. (2000). Chemical chaperones mediate increased secretion of mutant alpha 1-antitrypsin (alpha 1-AT) Z: A potential pharmacological strategy for prevention of liver injury and emphysema in alpha 1-AT deficiency. Proc. Natl. Acad. Sci. USA.

[26] Marcus N.Y., Perlmutter D.H. (2000). Glucosidase and Mannosidase Inhibitors Mediate Increased Secretion of Mutant 1 Antitrypsin Z. J. Boil. Chem..

[27] Sandhaus R.A. (2004). alpha(1)-antitrypsin deficiency center dot 6: New and emerging treatments for alpha(1)-anitrypsin deficiency. Thorax.

[28] Perteghella S., Bari E., Chlapanidas T., Sorlini M., De Girolamo L., Perucca Orfei C., Viganò M., Torre M.L. (2016). Process for isolating and lyophilizing extracellular vesicles.

[29] Bari E., Perteghella S., Catenacci L., Sorlini M., Croce S., Mantelli M., A Avanzini M., Sorrenti M., Torre M.L. (2019). Freeze-dried and GMP-compliant pharmaceuticals containing exosomes for acellular mesenchymal stromal cell immunomodulant therapy. Nanomedicine.

[30] Faustini M., Bucco M., Chlapanidas T., Lucconi G., Marazzi M., Tosca M.C., Gaetani P., Klinger M., Villani S., Ferretti V.V. (2010). Nonexpanded Mesenchymal Stem Cells for Regenerative Medicine: Yield in Stromal Vascular Fraction from Adipose Tissues. Tissue Eng. Part C: Methods.

[31] Gaetani P., Torre M.L., Klinger M., Faustini M., Crovato F., Bucco M., Marazzi M., Chlapanidas T., Levi D., Tancioni F. (2008). Adipose-Derived Stem Cell Therapy for Intervertebral Disc Regeneration: An In Vitro Reconstructed Tissue in Alginate Capsules. Tissue Eng. Part A.

[32] Dominici M., Le Blanc K., Mueller I., Slaper-Cortenbach I., Marini F.C., Krause D.S., Deans R.J., Keating A., Prockop D.J., Horwitz E.M. (2006). Minimal criteria for defining multipotent mesenchymal stromal cells. The International Society for Cellular Therapy position statement. Cytotherapy.

[33] Ferrarotti I., Scabini R., Campo I., Ottaviani S., Zorzetto M., Gorrini M., Luisetti M. (2007). Laboratory diagnosis of alpha1-antitrypsin deficiency. Transl. Res..

[34] Sinden N.J., Koura F., Stockley R.A. (2014). The significance of the F variant of alpha-1-antitrypsin and unique case report of a PiFF homozygote. BMC Pulm. Med..

[35] Bari E., Arciola C.R., Vigani B., Crivelli B., Moro P., Marrubini G., Sorrenti M., Catenacci L., Bruni G., Chlapanidas T. (2017). In Vitro Effectiveness of Microspheres Based on Silk Sericin and Chlorella vulgaris or *Arthrospira platensis* for Wound Healing Applications. Materials.

[36] Gorrini M., Ferrarotti I., Lupi A., Bosoni T., Mazzola P., Scabini R., Campo I., Zorzetto M., Novazi F., Luisetti M. (2006). Validation of a Rapid, Simple Method to Measure 1-Antitrypsin in Human Dried Blood Spots. Clin. Chem..

[37] Saito R., E Smoot M., Ono K., Ruscheinski J., Wang P.-L., Lotia S., Pico A.R., Bader G.D., Ideker T. (2012). A travel guide to Cytoscape plugins. Nat. Methods.

[38] Doncheva N.T., Morris J.H., Gorodkin J., Jensen L.J. (2019). Cytoscape StringApp: Network Analysis and Visualization of Proteomics Data. J. Proteome Res..

[39] Scardoni G., Tosadori G., Pratap S., Spoto F., Laudanna C. (2015). Finding the shortest path with PesCa: a tool for network reconstruction. F1000Research.

[40] Maere S., Heymans K., Kuiper M. (2005). BiNGO: A Cytoscape plugin to assess overrepresentation of Gene Ontology categories in Biological Networks. Bioinformatics.

[41] Scardoni G., Petterlini M., Laudanna C. (2009). Analyzing biological network parameters with CentiScaPe. Bioinformatics.

[42] Lara B., Martinez M.T., Blanco I., Hernández-Moro C., Velasco E.A., Ferrarotti I., Rodriguez-Frias F., Perez L., Vázquez I., Alonso J. (2014). Severe alpha-1 antitrypsin deficiency in composite heterozygotes inheriting a new splicing mutation QOMadrid. Respir. Res..

[43] Chlapanidas T., Faragò S., Lucconi G., Perteghella S., Galuzzi M., Mantelli M., Avanzini M.A., Tosca M.C., Marazzi M., Vigo D. (2013). Sericins exhibit ROS-scavenging, anti-tyrosinase, anti-elastase, and in vitro immunomodulatory activities. Int. J. Boil. Macromol..

[44] Anon B.S. (2001). EN 13697: 2001, Chemical Disinfectants and Antiseptics. Quantitative Non-Porous Surface Test for the Evaluation of Bacterial and/or Fungicidal Activity of Chemical Disinfectants Used in Food, Industrial, Domestic and Institutional Areas. Test Method and Requirements without Mechanical Action.

[45] Fraise A.P., Lambert P.A., Masillard J.-Y., Russell H.A.S. (2004). Principles and Practice of Disinfection, Preservation & Sterilization.

[46] Vella D., Zoppis I., Mauri G., Mauri P., Di Silvestre D. (2017). From protein-protein interactions to protein co-expression networks: a new perspective to evaluate large-scale proteomic data. EURASIP J. Bioinform. Syst. Boil..

[47] Rossi S., Marciello M., Sandri G., Ferrari F., Bonferoni M.C., Papetti A., Caramella C., Dacarro C., Grisoli P. (2007). Wound Dressings Based on Chitosans and Hyaluronic Acid for the Release of Chlorhexidine Diacetate in Skin Ulcer Therapy. Pharm. Dev. Technol..

[48] Amato E., Diaz-Fernandez Y.A., Taglietti A., Pallavicini P., Pasotti L., Cucca L., Milanese C., Grisoli P., Dacarro C., Fernandez-Hechavarria J.M. (2011). Synthesis, Characterization and Antibacterial Activity against Gram Positive and Gram Negative Bacteria of Biomimetically Coated Silver Nanoparticles. Langmuir.

[49] Baligar P., Kochat V., Arindkar S.K., Equbal Z., Mukherjee S., Patel S., Nagarajan P., Mohanty S., Teckman J.H., Mukhopadhyay A. (2017). Bone Marrow Stem Cell Therapy Partially Ameliorates Pathological Consequences in Livers of Mice Expressing Mutant Human a1-Antitrypsin. Hepatology.

[50] Vader P., Mol E.A., Pasterkamp G., Schiffelers R.M. (2016). Extracellular vesicles for drug delivery. Adv. Drug Deliv. Rev..

[51] Tsui N.B.Y., O Ng E.K., Lo Y.M.D. (2002). Stability of endogenous and added RNA in blood specimens, serum, and plasma. Clin. Chem..

[52] Alvarez-Erviti L., Seow Y., Yin H., Betts C., Lakhal S., A Wood M.J. (2011). Delivery of siRNA to the mouse brain by systemic injection of targeted exosomes. Nat. Biotechnol..

[53] Zhuang X., Zhang H.-G. (2012). Abstract 4831: Treatment of brain inflammatory diseases by delivering exosome encapsulated anti-inflammatory drugs from the nasal region to the brain. Exp. Mol. Ther..

[54] Kusuma G.D., Carthew J., Lim R., Frith J.E., Kusuma D.G.D., Carthew D.J., Frith D.J.E. (2017). Effect of the Microenvironment on Mesenchymal Stem Cell Paracrine Signaling: Opportunities to Engineer the Therapeutic Effect. Stem Cells Dev..

[55] Saparov A., Ogay V., Nurgozhin T., Jumabay M., Chen W.C.W. (2016). Preconditioning of Human Mesenchymal Stem Cells to Enhance Their Regulation of the Immune Response. Stem Cells Int..

[56] Noone C., Kihm A., English K., O’Dea S., Mahon B.P. (2013). IFN-γ Stimulated Human Umbilical-Tissue-Derived Cells Potently Suppress NK Activation and Resist NK-Mediated Cytotoxicity In Vitro. Stem Cells Dev..

[57] Crisostomo P.R., Wang Y., Markel T.A., Wang M., Lahm T., Meldrum D.R. (2008). Human mesenchymal stem cells stimulated by TNF-, LPS, or hypoxia produce growth factors by an NF B- but not JNK-dependent mechanism. Am. J. Physiol. Cell Physiol..

[58] Stoller J.K., Aboussouan L.S. (2012). A Review of alpha(1)-Antitrypsin Deficiency. Am. J. Respir. Crit. Care Med..

[59] Nelson D.L., Cox M.M. (2017). Lehninger Principles of Biochemistry.

[60] Alcayaga-Miranda F., Cuenca J., Khoury M. (2017). Antimicrobial Activity of Mesenchymal Stem Cells: Current Status and New Perspectives of Antimicrobial Peptide-Based Therapies. Front. Immunol..

[61] Lin L., Du L. (2018). The role of secreted factors in stem cells-mediated immune regulation. Cell. Immunol..

[62] Porzionato A., Zaramella P., Dedja A., Guidolin D., Van Wemmel K., Macchi V., Jurga M., Perilongo G., De Caro R., Baraldi E. (2019). Intratracheal administration of clinical-grade mesenchymal stem cell-derived extracellular vesicles reduces lung injury in a rat model of bronchopulmonary dysplasia. Am. J. Physiol. Cell. Mol. Physiol..

[63] Cosenza S., Toupet K., Maumus M., Luz-Crawford P., Blanc-Brude O., Jorgensen C., Noël D. (2018). Mesenchymal stem cells-derived exosomes are more immunosuppressive than microparticles in inflammatory arthritis. Theranostics.

[64] Di Trapani M., Bassi G., Midolo M., Gatti A., Takam P., Cassaro A., Carusone R., Adamo A., Krampera M. (2016). Differential and transferable modulatory effects of mesenchymal stromal cell-derived extracellular vesicles on t, b and nk cell functions. Haematologica.

[65] Sicco C.L., Reverberi D., Balbi C., Ulivi V., Principi E., Pascucci L., Becherini P., Bosco M.C., Varesio L., Franzin C. (2017). Mesenchymal Stem Cell-Derived Extracellular Vesicles as Mediators of Anti-Inflammatory Effects: Endorsement of Macrophage Polarization. Stem Cells Transl. Med..

[66] Taggart C., Mall M.A., Lalmanach G., Cataldo D., Ludwig A., Janciauskiene S., Heath N., Meiners S., Overall C.M., Schultz C. (2017). Protean proteases: at the cutting edge of lung diseases. Eur. Respir. J..

[67] Théry C A.S., Raposo G., Clayton A. (2006). Isolation and Characterization of Exosomes from Cell Culture Supernatants and Biological Fluids. Curr. Protoc. Cell Biol..

[68] Daviskas E., Anderson S.D., Young I.H. (2010). Effect of mannitol and repetitive coughing on the sputum properties in bronchiectasis. Respir. Med..

